# Next generation sequencing of Y-STRs in father-son pairs and comparison with traditional capillary electrophoresis

**DOI:** 10.1080/20961790.2021.1898078

**Published:** 2021-04-16

**Authors:** Steffi Bredemeyer, Lutz Roewer, Sascha Willuweit

**Affiliations:** Department of Forensic Genetics, Institute of Legal Medicine and Forensic Sciences, Charité – Universitätsmedizin Berlin, Berlin, Germany

**Keywords:** Forensic sciences, forensic genetics, Y-STRs, rapidly mutating Y-STRs, sequence polymorphism, isometric sequence variants, massively parallel sequencing

## Abstract

To evaluate the promising advantages of massively parallel sequencing (MPS) in our casework, we analysed a total of 33 Y-chromosomal short tandem repeats (Y-STRs) with traditional capillary electrophoresis (CE) and 25 Y-STRs using the newer MPS technology. We studied the outcome of both technologies in 64 father-son pairs using stock and custom-designed kits. Current MPS technology confirmed the 13 mutational events observed with CE and improved our understanding of the complex nature of STR mutations. By detecting isometric sequence variants between unrelated males, we show that sequencing Y-STRs using MPS can boost discrimination power.

## Introduction

Y-chromosomal short tandem repeats (Y-STRs) are especially helpful to analyse the male proportion of complex female—male mixtures, which is a typical scenario of samples in sexual assault cases. While autosomal STRs can easily be used to identify individuals, Y-STR profiles tend to be identical amongst patrilineal relatives because of their non-recombining inheritance mode along paternal lineages. Therefore, a male perpetrator most likely cannot be differentiated from his brother or father using Y-STRs [1]. While this feature of Y-STRs is detrimental when trying to differentiate paternally related individuals, it is beneficial for analysing deep-rooted pedigrees [2] and familial searching [3].

Currently, the standard technique used in forensic genetics is to type STRs with capillary electrophoresis (CE) analysis. This analysis method is fast (profiles can be generated within 1 day) and affordable for the average crime laboratory. Furthermore, the resulting data handling is straightforward, as STR length alleles are defined by the number of repetitive motifs by most standard software packages. This enables practitioners to report and compare results very easily. In contrast, massively parallel sequencing (MPS) is more time-consuming (at least 2 days hands-on in the laboratory and approximately 30 h sequencing time) and expensive, depending on how many samples are pooled per run [4]. However, MPS has some advantages compared with CE analysis: (1) the lack of specific shared fluorescent markers makes it possible to multiplex more than 100 markers in one reaction instead about 30 markers with CE analysis [5–7] and (2) MPS reveals the actual sequence of the variation rather than just its length, and is therefore able to detect sequence variants within alleles (isometric variants), which are not distinguishable with CE analysis. Sequence variants can appear as intra-repeat single nucleotide polymorphisms (SNPs), SNPs within the flanking region, indels, or repeat pattern variants. Using these sequence variants, two individuals could be distinguished with one marker even if they have the same nominal allele [8] and are thus indistinguishable using CE. Various studies have shown an increase in the number of alleles per locus when these isometric variants are analysed by MPS, which results in a higher genetic diversity of STRs [8–22]. Additionally, sequencing can cover flanking sequences and is thus able to detect SNPs that are not detecta­ble with CE [7,9,23,24]. However, SNPs have a rela­tively low mutation rate of 10^−8^ per base per generation [25] and are therefore unlikely to contribute to the separation of male relatives.

To determine if Y-STR sequence variants may help to improve the differentiation of paternally-related males in forensic casework, we compared the CE and MPS analysis methods based on mutations at 33 Y-STRs and 25 Y-STR markers of 64 father-son pairs.

## Materials and methods

### Sample preparation

We collected buccal swap samples from 64 father-son pairs from European populations with written informed consent signed by all participants. Our analyses are based on self-declared family relations. DNA extraction was performed on the BioRobot® EZ1 (Qiagen, Hilden, Germany) using the EZ1 DNA Blood 200 µL Kit (Qiagen). Quantification of DNA samples was performed with the Rotor-Gene Q (Qiagen) and the Investigator Quantiplex Kit (Qiagen) or with the Quantus^™^ Fluorometer (Promega Corporation, Madison, WI, USA) and the QuantiFluor^®^ ONE dsDNA Kit (Promega Corporation).

#### CE analysis

A total of 33 Y-STR markers were amplified by combining 22 Y-STR markers of the PowerPlex^®^ Y23 System (Promega Corporation) and 11 Y-STR markers of an adapted RM-Yplex assay (13 Y-STRs in total) based on Alghafri et al. [26]. The modified primer mix can be found in Supplementary File S1. Instead of the DYS518 marker that was used by Alfghafri et al. [26], the marker DYS464 was added to the multiplex PCR. This multi-copy Y-STR marker with at least four copies on the Y chromosome was selected for its outstanding diversity [27,28]. The primer sequence for DYS464 was taken from Redd et al. [29] and labelled with ATTO 550. PCR amplification was performed in a final volume of 15 µL per reaction: 7.5 µL Platinum™ Multiplex PCR Master Mix (Thermo Fisher Scientific, Waltham, MA, USA), 3.3 µL primer mix according to Supplementary File S1, 3.2 µL PCR grade water, and 1 µL DNA template (1 ng/µL). To confirm a duplication observed within the marker Y-GATA-H4 in one sample we performed an additional PCR using the Yfiler™ Plus PCR Amplification Kit (Thermo Fisher Scientific) with this sample and the sample of the associated son. CE ana­lysis was carried out on the 3500 Genetic Analyzer (Applied Biosystems, Forster City, CA, USA) and data were analysed using the GeneMapper IDX version 1.4 software (Thermo Fisher Scientific).

#### MPS analysis

Library preparation for sequencing analysis was performed with the ForenSeq^™^ DNA Signature Prep Kit (Verogen, San Diego, CA, USA) according to the manufacturer’s instructions. For PCR amplification, primer mix A was used, which includes 25 Y-STR markers. All samples were sequenced with the MiSeq FGx (Illumina, San Diego, CA, USA) using the MiSeq FGx Reagent Kit (Verogen). For better coverage, the library input volume was increased from 7 µL to 10 µL. Data analysis was done using an in-house analysis pipeline (as explained below), allowing us to analyse the marker DYS456, which is included in the primer mix of the ForenSeq^™^ DNA Signature Prep Kit but excluded from the ForenSeq^™^ Universal Analysis Software (UAS, Verogen). Allele-stutter differentiation was done following the forward-, back-, and double-back-stutter models given by Bright et al. [30] and the vendor-provided expected stutter percentages (same for UAS and the in-house pipeline). All results with less than 10 reads were discarded.

### In-house analysis pipeline

To be able to assess the analysis done by the UAS software, we re-analysed the raw FASTQ files using a bioinformatic pipeline called “wintermute” (open-source software available online at https://github.com/545ch4/wintermute, publication in preparation). This tool was prototyped as part of the EU funded DNASeqEx (DNA-STR Massive Sequencing & International Information Exchange) project, which aimed to enable the user to understand the variability and composition of the huge number of single reads/sequences within an MPS FASTQ file. In the first step, the software assigns each read to one or more targets (derived from the target primer that was used). All sequences of those sequence-buckets are then multi-aligned to generate one or more consensus sequence that represents the sequence-bucket. The software does not generate reliable results for the flanking regions of the Y-STRs used here. As the sequence quality declines over read length (approx. beginning at 120 bp), the software was unable to generate merged flanking sequences of longer Y-STR (e.g. DYS448 and DYS389II). Therefore, we decided to exclude all flanking sequences for reasons of consistency.

## Results

We used CE analysis to examine 22 Y-STR markers of 64 father-son pairs using the PowerPlex^®^ Y23 System and used MPS analysis for 25 Y-STRs of the same sample set using the ForenSeq^™^ DNA Signature Prep Kit. DYS393 and DYS458 are included in the PowerPlex^®^ Y23 System, but not in the ForenSeq^™^ DNA Signature Prep Kit. DYF387S1, DYS460, DYS505, DYS522, and DYS612 are included in the ForenSeq^™^ DNA Signature Prep Kit, but not in the PowerPlex^®^ Y23 System. In total, 20 Y-STR markers were analysed using both kits (Supplementary File S2). Comparing MPS results with CE results, all allele designations were concordant with the exception of one drop-out (in DYS392) because of low coverage of this sample. There were some stutters above the stutter filter, namely in DYS385ab, DYS392, DYS456, and DYS576, and some additional sequence variants (drop-ins) slightly above a peak height ratio (PHR) of 10% in DYS437 and DYS448. All drop-ins were below a PHR of 13% and were therefore not calculated as “real” alleles. Notably, the two alleles of the DYS385ab marker were sometimes unbalanced in the MPS analysis (minimum 21.5% PHR for DYS385b). Additionally, a duplication at Y-GATA-H4 in one sample was observed with both CE and MPS (MPS: 495 reads, 76% PHR for the CE allele 10).

Furthermore, we used CE to examine 13 rapidly mutated Y-STRs using a modified RM-Yplex assay developed by Alghafri et al. [26] (including DYS464 in place of DYS518) of the same sample set. DYF399S1, DYF403S1, DYF404S1, DYS449, DYS464, DYS526, DYS547, DYS626, and DYS627 are included in the RM-Yplex assay, but not in the ForenSeq^™^ DNA Signature Prep Kit. DYF387S1, DYS570, DYS576, and DYS612 are included in both the RM-Yplex assay and ForenSeq^™^ DNA Signature Prep Kit (Supplementary File S2). Similar to our previous results, all allele desi­gnations were concordant, except some stutters above the stutter filter in DYF387S1 and DYS612.

In total, 13 mutations were detected when analysing 22 Y-STR markers of 64 father-son pairs with CE using the PowerPlex^®^ Y23 System (Supplementary File S2): two events at marker DYS19, two events at DYS448, and one event each at DYS385ab, DYS389I, DYS389II, DYS456, DYS458, DYS481, DYS549, DYS643, and Y-GATA-H4. All mutations were either one-repeat losses or one-repeat gains, with the exception of the mutational event at Y-GATA-H4, which was caused by a duplication. Mutational events occurred at markers with simple repeat structures (DYS385ab, DYS456, DYS458, DYS481, DYS549, DYS643, Y-GATA-H4), at markers with complex repeat structures (DYS389I, DYS389II, DYS448), and at one marker with a compound repeat structure (DYS19). They were observed in sequences with a trimeric repeat structure (DYS481), a tetrameric repeat structure (DYS19, DYS385ab, DYS389I, DYS389II, DYS456, DYS458, Y-GATA-H4), a pentameric repeat structure (DYS643), and a hexa­meric repeat structure (DYS448).

Using MPS analysis (ForenSeq^™^ DNA Signature Prep Kit, 27 Y-STR loci) we confirmed 12 of 13 mutational events in the same cohort (DYS19, DYS448, DYS385ab, DYS389I, DYS389II, DYS456, DYS481, DYS549, DYS643, and Y-GATA-H4; Supplementary File S2). Two mutations at DYS458 and DYS612 could not be confirmed because of the absence of these markers either in the ForenSeq^™^ DNA Signature Prep Kit or the PowerPlex^®^ Y23 System. All mutations observed with MPS analysis were length-based mutations with the exception of the duplication at Y-GATA-H4 mentioned earlier. No additional sequence-based mutations were found. However, by sequencing these Y-STRs, we were able to determine the specific part of the repetitive stretch that causes the length variation: DYS19—the first repeat (of two compound repeats), DYS389I—the first repeat (of two compound repeats), DYS389II—the third repeat (of four compound repeats), DYS448—the first repeat (of three compound repeats), and DYS612—the third repeat (of three compound repeats) (Supplementary File S2). All mutational events occurred at the longest uninterrupted repeat sequence (LUS) [31].

By analysing the same sample set with our modi­fied RM-Yplex assay (13 Y-STR markers with up to 24 Y-STR alleles), we identified eight mutational events (Supplementary File S2) and were able to confirm the mutational event at DYS612 found with the ForenSeq^™^ DNA Signature Prep Kit. All remaining seven mutations occurred at markers not included in the PowerPlex^®^ Y23 System nor in the ForenSeq^™^ DNA Signature Prep Kit: two at DYF399S1, three at DYF403S1, one at DYF404S1, and one at DYS526. Each of these markers, categorized as RM Y-STR with an average mutation rate of 10^−2^ per locus per generation [32], belongs to the Y-STR multicopy marker and contains a complex repeat structure (Supplementary File S2). All observed mutations showed either one-repeat losses or one-repeat gains.

**Figure 1.**
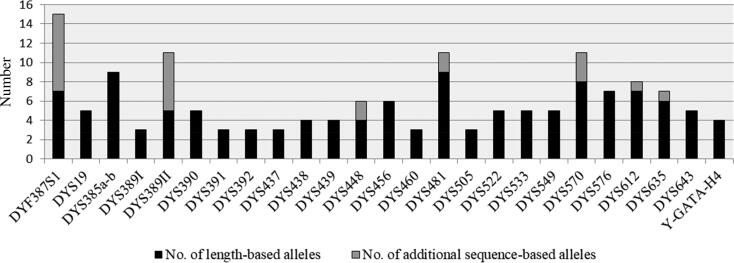
Observed allele increase with massively parallel sequencing (MPS) analysis using the ForenSeq^™^ DNA Signature Prep Kit (over 29 families).

We calculated the number of alleles per marker for CE analysis (PowerPlex^®^ Y23 System) and for MPS analysis (ForenSeq^™^ DNA Signature Prep Kit) by looking at all 29 families combined (Supplementary File S2) and identified 112 alleles in our CE analysis results and 151 alleles in our MPS analysis results. Of these 151 alleles detected by MPS, 128 alleles were length-based alleles, which are detectable with CE and MPS in the same manner. The remaining 23 isometric alleles were found to be different based only on their sequence, as they were not detectable with CE: eight at DYF387S1, six at DYS389II, two each at DYS448 and DYS481, three at DYS570, and one each at DYS612 and DYS635 (Figure 1, Supplementary File S2). Most of these markers were defined by a complex repeat structure (DYF387S1, DYS389II, DYS448, DYS612, DYS637), whereas only two of them by a simple repeat structure (DYS481, DYS570).

When looking for the specific part of the repeti­tive stretch that caused the length variation, we found that more repeats than only the LUS tended to vary in complex markers: two repeats each in DYF387S1 and DYS448, three repeats in DYS389II, and one repeat (LUS) in DYS635. Additionally, alleles longer than 23 (CE length-based) at DYS635 carried an insertion of [TACA]2[TAGA]4 (Supplementary File S2).

## Discussion

In summary, we identified the same 13 mutational events in 64 father-son pairs with CE analysis (PowerPlex^®^ Y23 System) and with MPS analysis. Compared with traditional CE analysis, we were unable to differentiate more father-son pairs with MPS analysis than with CE using the PowerPlex^®^ Y23 System. All mutational events detected by MPS were length-based variants, which is in concordance with CE analysis.

Mutations were observed at all types of markers, including simple and complex repeat structures and trimeric to hexameric repeat structures. Our results suggest that tetranucleotide repeats are more prone to mutations than other motifs [32–34], mutation events are mostly restricted to the LUS of a compound/complex STR marker [31,32,34,35], and the LUS length correlates with the mutability of an STR marker [32–34,36–38].

Unsurprisingly, when looking at the sequence variants between unrelated males, we found that the observed length variance is mainly explained by changes in the repetitive structure of a Y-STR. Hence, Just and Irwin et al. [31] showed that the combination of a length-based allele and LUS length as a designator can represent more than 80% of the variability detected by sequencing. Our data concur with their observations, as nearly all isometric sequence variants could be transformed using this method without losing any information. Only one exception was found at marker DYS389II, where four different sequence variants (CE length-based allele 30) would be denoted as two alleles (two times CE allele 30 + LUS allele 11 and two times CE allele 30 +LUS allele 12). This finding may help improve existing simplified mutation models (SMM) and phylogenetical separation used in population genetics.

Our results are in agreement with earlier findings [9,39,40] that isometric sequence variants mostly occur at complex STR markers. The largest increases for additional sequence-based alleles were found at DYF387S1 (consistent with [9,41]) and DYS389II (consistent with [39,41–43]). This could be very important for deep-rooted pedigree and paternity analysis using Y-STRs because specific knowledge of the mutation event could facilitate the comparison of more complex mutation models to the simplified single step mutation model [44].

As Huszar et al. [39] predicted, we could not find any SNP or indel because of the associated lower mutation rates of these mutational events. We therefore agree with their conclusion that male identification using Y-STRs may not be greatly advanced by applying forensic MPS approaches [39]. In our MPS analysis, we found the same number of mutational events as with CE analysis. However, when analysing the dataset with RM Y-STRs (CE), we found eight additional mutations. Thus, for diffe­rentiation of father-son pairs, analysing more RM Y-STRs with CE seems to be a much more promising approach than just sequencing the common Y-STRs. Recently, Ralf et al. [34] proposed 12 new RM Y-STRs with a mean mutation rate of 2.6 × 10^−2^, demonstrating high discrimination power. We also agree with Huszar et al. [39] that applying MPS to RM Y-STRs is expected to increase discriminatory power as allele diversity increases. However, MPS analysis using the ForenSeq^™^ DNA Signature Prep Kit includes the results of autosomal STRs, Y-STRs, X-STRs, and seve­ral SNPs at the same time. This would allow for easy individualization, except in female–male DNA mixtures where the male proportion is masked by the female component.

Though we did not find any additional sequence-based mutations between fathers and sons, we found many additional sequence-based alleles between unrelated males. These isometric variants are helpful to exclude a suspect or to deconvolute mixtures of multiple male contributors [39,43], and to detect private or rare mutations pointing to a remote patrilinear relation.

One major drawback of MPS is the amount of novel and challenging bioinformatics methodology necessary for analysing new data. When restricted to vendor-provided tools and protocols, researchers may miss important new features of this technology. Besides basic measurements like sequence reads, cove­rage, and quality assessment, the major obstacle is a missing internationally standardized nomenclature [45], which would enable laboratories to exchange MPS data without an unnecessary reduction to CE alleles. In summary, we believe that Y-STR MPS ana­lysis is much more demanding for the forensic crime laboratory than traditional CE analysis. Together with the much higher cost of MPS, this could explain the rather hesitative adoption of this new technology in the forensic genetics field. CE will likely remain the standard method for genetic profiling for the next several years, even though especially at Y-STRs MPS could reveal much more information and help to understand the mechanisms of mutational events beyond simplified models like SMM [44].

## Supplementary Material

Supplemental Material
